# Supplementary material for: Changes-of-mind in the absence of new post-decision evidence
Source: PLoS Comput Biol. 2020 Feb 3;16(2):e1007149. doi: 10.1371/journal.pcbi.1007149 (PMC7018100; doi:10.1371/journal.pcbi.1007149)
Supplement: S1 Appendix — (PDF) [file pcbi.1007149.s001.pdf]

S1 Appendix

Analysis of the extended drift-diffusion model

In the extended drift-diffusion model (eDDM) developed in [1], the dynamics of evidence accumulation can be described by:

$$dx = dt(kc + \mu_0) + \sqrt{dt}\sigma \tag{1}$$

where:
x: Dynamical (decision) variable.
c: Coherence level.
k, μ_0: Free parameters that control the speed-accuracy trade-off
σ: A random variable sampled from a normal distribution, i.e. $\sigma \sim \mathcal{N}(0, 1)$.

In the eDDM, the initial decision and decision time are determined by the threshold B (or $-B$). However, after the initial decision, evidence accumulation continues until threshold B_Δ is reached for confirmation of the initial decision or a change-of-mind. In particular, the post-decision accumulation process is driven by late-incoming evidence (and noise fluctuations), with a change-of-mind deadline of around 300ms. eDDM has been previously used to fit response times (and choice accuracy) from change-of-mind trials in reaction-time tasks, in which stimulus offset and response onset coincided [1, 2]. The eDDM suggested, consistent with the experimental data in such paradigms [1, 3], that change-of-mind trials are most likely to occur during trials with faster response times.

To provide an insight into the eDDM predictions regarding change-of-mind behaviour in the context of the current study, we simulated the eDDM with the stimulus input $kc+\mu_0$ set to 0 immediately after the initial decision (using other parameter values that are fitted to the data from Subject S in [1]). This setup mimics the situations where no additional evidence is available after the initial decisions. Because eDDM does not incorporate any additional assumptions about other processes potentially inducing changes-of-mind [1], the post-decision process in this setup is driven solely by noise fluctuations. Not surprisingly, we found that in this setup, eDDM does not account for the observed change-of-mind behaviour. Specifically, the majority of changes-of-mind in this case were correct-to-error changes, with increasing frequency of changes-of-mind with coherence level (Fig. A). Furthermore, the majority of change-of-mind trials at high coherence levels had fast initial response times (Fig. A).

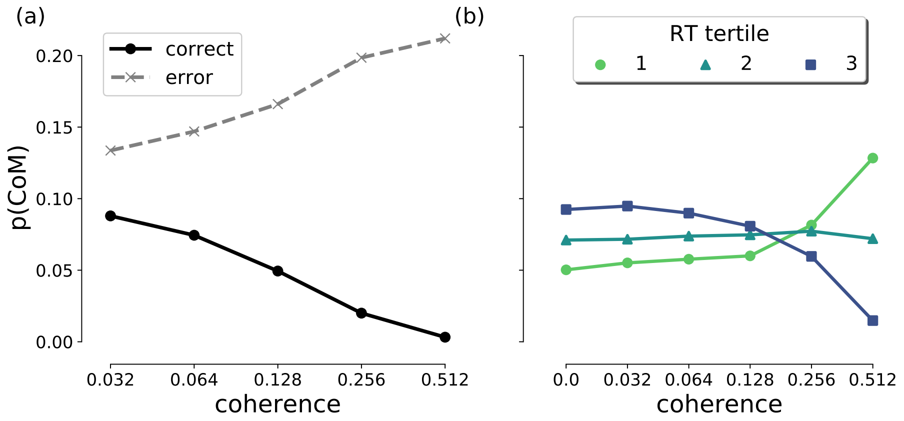

Fig. A: Results of simulating eDDM in the scenario of stimulus discontinued immediately after the initial decision. **(a)** When the post-initiation process is driven entirely by noise fluctuations, the majority of changes-of-mind are errors. **(b)** At high coherence levels (i.e. ≥ 0.256), the majority of changes-of-mind occur in trials with fast initial response times.

Supplementary References

[1] Resulaj A, Kiani R, Wolpert DM, Shadlen MN. Changes of mind in decision-making. Nature. 2009;461(7261):263–266. doi:10.1038/nature08275.

[2] Van Den Berg R, Anandalingam K, Zylberberg A, Kiani R, Shadlen MN, Wolpert DM. A common mechanism underlies changes of mind about decisions and confidence. eLife. 2016;5:e12192.

[3] Albantakis L, Branzi FM, Costa A, Deco G. A multiple-choice task with changes of mind. PLoS ONE. 2012;7(8). doi:10.1371/journal.pone.0043131.
