# Supplementary material for: Changes-of-mind in the absence of new post-decision evidence
Source: PLoS Comput Biol. 2020 Feb 3;16(2):e1007149. doi: 10.1371/journal.pcbi.1007149 (PMC7018100; doi:10.1371/journal.pcbi.1007149)
Supplement: Attachment — Submitted filename: response_to_reviewers.pdf [file pcbi.1007149.s002.pdf]

Response to reviewers

Review #1

In this very interesting paper, Atiya et al analyze the changes of mind (CoM) that occur in the absence of new post-decision information. Previous papers on CoM have invariably used paradigms where new evidence arrives after the decision has already been made. Therefore, the CoMs observed in those studies are easily interpreted as simply the newest evidence being stronger than the older one, which makes it a much less interesting phenomenon. The authors here make a significant advance by showing that CoMs occur even in the absence of new evidence. However, critically, the signatures associated with these CoMs are very different than the signatures in previous studies with different designs. These are important discoveries. I only have major suggestions regarding the presentations of the results.

We thank the reviewer for appreciating our work.

My only major comment is that, as it stands, the paper is rather difficult (and sometimes even frustrating to read). There are a number of reasons. Several sections start with a number of assertions in the first paragraph that are only later supported by stats in the second paragraph. I'd suggest introducing the stats the first time a claim is made; otherwise, the reader has to keep in working memory all claims that were previously made and check whether there's support for them only later. The Results section starts with a heading titled "Cognitive task and neural circuit model". That section includes some background and a single "teaser" result followed by introducing the model when it was not yet clear what needs explaining (the 3% CoM alone could certainly be explained in many other ways).

More generally, the organization of the manuscript makes it seem (at least for a while) that it's mostly about the authors' model. However, I think that the strongest part of this paper is the behavioral results, which become somewhat secondary in the current writeup. I'd suggest changing the organization where the behavioral effects are presented first, followed by the eDDM results, and only then followed by the authors' model. This would also highlight that the model can fit patterns of results that are not "easy" to fit as eDDM cannot fit them.

We appreciate the reviewer's comments regarding the organisation of the manuscript. We have made major changes to the flow of the manuscript. The main manuscript now first presents a description of the experimental task and behavioural results, which is then followed by neural circuit modelling descriptions and results. We decided to place the eDDM results in the Supporting Information and not in the main manuscript specifically to make the manuscript more focused on the novel results. The eDDM was not designed to capture changes-of-mind without additional post-decision evidence (this limitation was already discussed in Resulaj et al., 2009); for this reason, we do not want to emphasize the comparison between our model and eDDM. Further, in the revised manuscript we provide the statistical support right after stating each experimental result.

1. It was rather frustrating to read this paper as the figures were placed at the end (journals should not require this and if they do, authors should ignore it for the first submission in the interest of everybody) and figure legends appeared before the figures were references in the

main text. I assume that none of this is the authors' choice but I suggest re-formatting future submissions.

We agree with the reviewer. We have now embedded the figures into the text for better readability.

2. P.5 "However, in change-of-mind trials, choice accuracy was on average lower than in non-change-of-mind trials." This statement appears to me to be completely unrelated to the one above (why should the trends in these two analyses be related?) but the use of the word "however" suggests otherwise.

We thank the reviewer for this comment. We have now changed the statement to (Results Section, page 4): "*In change-of-mind trials, choice accuracy was on average lower than in non-change-of-mind trials (b=-1.3, z=-11.9, p<0.0001).*"

3. P.5 "However, although there was an ascending trend for the rate of correct changes-of-mind with increasing coherence level, the trend was not as steep as that observed in the experiment." Some sort of statistical analysis would help here.

Following the suggestions of the reviewers, we have changed the structure of the Results section, and in the current revision of the paper we no longer contrast effect sizes from the model and the experiment. Instead, we emphasize the experimental findings and the fact that the model provides a qualitative account for these findings.

4. P.5 "followed the '<' pattern" – may be clearer to spell this out.

We thank the reviewer for the suggestion – this is now rephrased as (in the Results section, page 3): "*For correct choices, initial response times were on average faster than in error choices (b=-0.12, t=-20.0, p<0.0001). There was no evidence for the main effect of coherence (b=0.07, t=0.4, p=0.7); however, we found significant interaction between coherence and choice correctness (b=-0.6, t=-13.3, p<0.0001). These results indicate that response time decreased with coherence, but only in correct trials, which is consistent with previous perceptual discrimination studies using the reaction-time task (Shadlen & Newsome, 2001; Resulaj et al., 2009)*"

5. How are the 294 CoMs distributed among the 4 subjects?

We have now mentioned this in the Results section (page 3):
"we observed changes-of-mind in 3% trials (0.3% to 6.1%, median 2.4% across 11 participants)"
Note that while addressing Reviewer 2's concerns, seven additional participants have been recruited, resulting in a total of N=11, and 775 CoMs across all participants.

6. I may have missed this but how was a CoM defined exactly? What is the cutoff for determining that subjects moved sufficiently towards one target before changing? More speculatively, given that the authors have continuous measurements, can CoM itself also be

defined as a graded variable (I'm not suggesting that the authors do this but it may be worth discussing the possibility)?

The definition of CoM is clarified in the Data analysis subsection of the Methods and Materials section: *"A trial was labelled as a change-of-mind if a response trajectory deviated from the (implicit) vertical centre line towards the unchosen option by more than 100 pixels (in the x-direction). However, deviations which could have resulted from erratic movements in the early stages of response were ignored: if the threshold of 100 pixels in the horizontal direction was crossed in the bottom 10% of the response area, the trial was not labelled a change-of-mind."* (See page 15). The value of 100 pixels was determined by visual inspection of boundary cases (50 to 150 pixels). We verified the robustness of our analyses using a less conservative threshold of 50 pixels; this increased the number of CoMs by 30% but did not affect our conclusions.
In response to a more speculative comment that some kind of graded measure of a change-of-mind could be useful, we agree that this is a very interesting question. However, due to the fact that a change-of-mind can be characterized by many graded variables (including time of CoM, position on the screen where CoM happens, the speed of movement following a CoM and other potentially interesting measures) we prefer to leave this investigation for future work.

7. I commend the authors for sharing their data and codes. They write that "All codes and data needed to evaluate or reproduce the figures and analysis described in the paper are available online at: https://osf.io/y385t/. This includes the collected data, and the code for stimulus presentation, model simulation, and data analysis." However, when I followed the link, I didn't find any codes – it appeared that what is there is only raw(-ish) data. I also suggest including a file that provides a key to what is contained in each folder as well as a license document (typically CC-BY is a good idea).

We thank the reviewer for appreciating our commitment to open science, and we apologise for the inconvenience while attempting to access our code and data. We have now made sure that the URL https://osf.io/y385t/ leads to the correct code repository and data source. We now also include the license and added a data dictionary.

Review #2

The manuscript examines the phenomenon of change of mind (COM) in perceptual decisions with moving dots stimuli, and with responses that are required after stimulus offset. This differs from some previous studies that examined COM responses in a situation where response terminate the stimulus. The manuscript reports data from 4 participants, which shows about 3% COM responses, and which appear to increase with stimulus difficulty (low coherence) and with RT (more COM at slow responses). These two pattern are different from what was reported in conditions where the response terminates the stimulus, and the authors propose a computational model based on uncertainty monitoring (rather than integration of extra-evidence), which accounts for qualitative aspects of the data.

While I find the question – do COM require extra-evidence and if not what mechanism can account for them – interesting, and the work carried out of good professional standard, there are three important reasons why I do not feel that, this paper is ready for publication in this

Journal. First, the experiment includes only 4 participants. Second the literature discussed in quite incomplete, and finally, the paper is difficult to read and at many places the Method and the Computational mechanism proposed is not clear (and appears somewhat adhoc). Below I elaborate on these 3 points before I provide a number of more specific comments, which the authors should address in a potential revision.

1. participants

4-participants is really below the standard in cognitive psychology (a set of 8-12 would be a minimum requirement). While psycho-physical studies do sometimes uses such small designs, this is usually done when each subject is tested over repeated sessions, and individual data are presented (showing the same pattern in each participant). This is definitely not the case here, especially as the critical COM trials are quite rare (<3%).

To address the reviewer's comment regarding the sample size, we have now added 7 new participants, resulting in N=11. As the reviewer has rightly noted, changes-of-mind are rare, which makes it difficult to analyse the results for each individual participant. However, our analyses indicate that all findings of the initial submission (N=4) were replicated in this new sample (N=7). For this reason, we treat all 11 participants as a single sample in the revised manuscript.

2. Literature.

There is much literature, both experimental and computational, which seems quite related to the issues discussed here, which is neither mentioned nor discussed. On the experimental side there are numerous papers by Rabbitt P. and colleagues, on error-correction (or double responding), which seems to me to probe a quite similar phenomenon to the one discussed in this paper (see also study by Bronfman et al., 2015, Exp-2, which reports more correct than incorrect COM). Even if those did not use moving dots stimuli, it will help to discuss differences (or commonalities) in the experimental results and in the mechanisms that were proposed. Second, there are computational models that extended the drift-diffusion model framework to competitive interactions (e.g., Bogacz et al. 2006), and to conflict monitoring (Botvinick et al., 2001). The latter, in particular, is somewhat similar to the uncertainty mechanism proposed here (except that it is multiplicative rather than additive). Since conflict and uncertainty seem related, a discussion of differences would be much needed (for example are there important differences between multiplicative and additive mechanisms, and which is more plausible?) A list of relevant references is provided below.

We thank the reviewer for pointing out these relevant references. We have now discussed most of the suggested references in the Introduction and Discussion sections of the revised manuscript as described below.

- On error correction and how it relates to changes-of-mind:
  Introduction, end of 1st paragraph:
  "Such reversals of decisions in response to new evidence have also been previously linked to error correction (Rabbitt, 1966; Rabbitt, 1967; Rabbitt et al., 1978; Rabbitt &

Vyas, 1981), indicating a strong association between the mechanisms underlying these behaviours." (page 2)

Discussion, 2nd paragraph:

"Early modelling and experimental work on error correction (Rabbitt, 1966; Rabbitt, 1967; Rabbitt et al., 1978; Rabbitt & Vyas, 1981) suggested that error correction can be characterised by an evidence accumulation process in which initial error responses are reversed (or corrected) by new incoming evidence that negates the initial (erroneous) judgement. Hence, current experimental investigations of changes-of-mind lie within the same framework, reinforcing the link between error correction and changes-of-mind." (pages 12-13)

- On how our model relates to other simpler models:

  Discussion, 5th paragraph:

  "The model proposed here is a variant of the previously developed model (Atiya et al., 2019), which was inspired by neurophysiological recordings from brain regions encoding decision confidence or, reciprocally, decision uncertainty (Kepecs et al., 2008; Murphy et al., 2015). In this work, we have reduced the uncertainty-monitoring module of the original model to lay bare its essential functions. In addition to the similarity of the neural profile of our model's uncertainty-encoding population (Figure 6) to existing recordings (Kepecs et al., 2008; Murphy et al., 2015), this reduced model accounts for some of the main characteristics of decision uncertainty (Kepecs et al., 2008; Kiani et al., 2014; Sanders et al., 2016) (Figure 7). It should be noted that this reduced implementation of decision uncertainty could arguably be less neurobiologically plausible compared to the previous model, as the latter involves a canonical cortical column structure - inhibitory-excitatory pair of neural populations (Wilson & Cowan, 1972; Douglas & Martin, 2004). Importantly, we have not changed how the other (i.e. sensorimotor and motor) modules are described, which can serve as further validation of previous modelling work (Atiya et al., 2019). In particular, the sensorimotor module is based on a previously developed mean-field model with biologically derived variables (Wong & Wang, 2006). Therefore, the model can be tested in future studies using neurophysiological recordings within similar experimental task paradigms to allow the distinguishing between different models (O'Connell et al., 2018). It should also be noted that such reductions of biophysical realisations of accumulate-to-bound models (Wang, 2012) can be linked back to simpler models of decision making (Ratcliff, 1978; Usher & McLelland, 2001; Bogacz et al., 2006; Wong & Wang, 2006; Roxin & Ledberg, 2008)."

- Discussing conflict monitoring:

  Discussion, second last paragraph:

  "Decision uncertainty or confidence is closely related to (decision) conflict monitoring. Computational models of cognitive control have used conflict monitoring to account for various behavioural aspects of decision uncertainty (Botvinick et al., 2001). For instance, such models have been shown to account for post-error slowing, attentional bias, error-related negativity, error-prediction, and neuromodulatory processing (Yeung et al., 2004; Greene et al., 2004; Kerns et al., 2004; Gao, Wong-Lin et al., 200x9; Alexander & Brown, 2011). In conflict monitoring models, conflict is usually modelled by a dedicated conflict monitoring unit that takes as an input the instantaneous

activities of the competing (e.g. decision) units, with the output being the multiplication of these activation rates. This representation accounts for various signatures of conflict. Specifically, conflict typically increases with increasing activities of the competing units, and conflict level is highest (maximal) level when competing units reach their maximal activities. Unlike these conflict-monitoring models, which are mainly based on abstract sigmoidal- or logistic-like activation (input-output) functions, the original decision uncertainty module (Atiya et al., 2019) is inspired by a canonical cortical microcircuit model (Wilson & Cowan, 1972; Douglas & Martin, 2004), which the current model is reduced from. Further, its input-output function is represented by a simple threshold-linear model, i.e. neural activity saturation is unnecessary. Importantly, the quantification of decision uncertainty involves the more biologically plausible mechanism of summation of inputs from presynaptic neurons (Dayan and Abbot, 2001), as compared to the multiplication of inputs in conflict-monitoring models. Moreover, our model mechanistically relates the decision uncertainty to continuous motor output dynamics."

3. Computational Model

I found it quite difficult to understand the model, which could be partly due to the very compressed writing in the main text. As I don't think that this journal has a serious limitation on the number of words, I would propose to the authors to provide systematic and clear descriptions of the model, in the main text, rather than asking the readers to work hard by shifting back and forth to the Method or Supplement sections. More important, it appears to me that many of the modeling assumptions are somewhat adhoc. Just to give one example, while the sensorymotor populations are described by a "reduced spiking model" (Eq. 5-9), the hand-populations (eq. 10-11) and the uncertainty population are described by more simple firing-rate equations. As the present paper does not address any neural data, I find this variability unmotivated and somewhat distracting. Thus I would suggest to the authors to rely on the same dynamics throughout the paper (I would prefer the simpler ones; 10-11), unless there is an important reason why this cannot be done. Also, there are at present 13 model parameters and it is not very clear why they are picked as they are (was there any data fitting?)

We appreciate this comment, and we apologise for the difficulties encountered during reading our manuscript. We have now addressed this by changing the flow of the manuscript. This is done by first describing the experimental task and behavioural results, which is then followed by neural circuit modelling descriptions and results. We decided to place the eDDM results in the Supporting Information and not in the main manuscript specifically to make the manuscript more focused on the novel results. Furthermore, we expanded the explanation of the model in the Results section and added relevant comparisons with other conflict-monitoring models (in response to previous comments) in the Discussion section.

Specifically, in response to the reviewer's comment regarding the use of different equations to describe the modules of the model, we have referred the readers to the Methods and Materials section given that the model has already been introduced in Atiya et al. (2019). Nevertheless, we have provided a general description of the model by adding the following paragraphs in the Results subsection "Neural circuit model" (paragraph 2):

“Here, we propose a version of the model which simplifies the neural circuit architecture developed in Atiya et al. (2019) (see Methods and Materials). Specifically, we used only one neuronal population to encode decision uncertainty, termed the uncertainty-monitoring population (Figure 3a, red circle). We simulated this model (Figure 6) Importantly, the phasic nature of the neural activity of the uncertainty-monitoring population (Figure 3b, red activity traces) is reminiscent of neural recordings from regions or neurons that encode decision uncertainty (Kepecs et al., 2008; Murphy et al., 2005). In our model, 600 ms after stimulus onset, this uncertainty-monitoring population receives and integrates the summed neural activities of the sensorimotor populations (Figure 3a, two-way red arrows), therefore continuously monitoring decision uncertainty during decision making. The uncertainty-monitoring population then in turn equally excites both sensorimotor neuronal populations, effectively providing them with excitatory feedback. When the activity of one of the sensorimotor populations reaches a prescribed threshold (42.5 Hz), the uncertainty-monitoring population stops integrating the summed input from the sensorimotor populations. We observed this equal excitation to be maximal in trials with error choices, during difficult tasks, and change-of-mind trials. This is due to the increased absolute input to the uncertainty-monitoring populations under such conditions (Figure 3a), in which sufficient time is allowed for the uncertainty-monitoring population to integrate the input (see also Atiya et al., 2019).

In quantifying the model’s uncertainty as a function of coherence level, we found that uncertainty level increases with coherence for error choices, but decreases for correct choices (Figure 7), which is consistent with previous findings (Sanders et al., 2016; Kepecs et al., 2008; Fleming et al., 2017). Furthermore, we found that this positive uncertainty feedback loop is strongly associated with initial response times (Pearson’s r = 0.74), similar to previous experimental work on decision uncertainty (Kiani et al., 2014). Taken together, these observations on the relationship between uncertainty, task difficulty, and response time can serve as a basis for explaining the underlying mechanism for changes-of-mind observed in our experimental task. However, in what follows, we first show that the model qualitatively accounts for the behavioural effects observed in our experiment.”

With regards to the suggestion of implementing the same type of dynamics, it should be noted that this model is based on our previously published computational model (Atiya et al., 2019), which were in turn built on top of the biophysical model in Wong and Wang (2006). In this work, we reduced the uncertainty monitoring module to reveal its key functions, while addressing the dynamics of hand movements of our experimental data. All other parameters were the same as in Atiya et al. (2019). It is true that we could have used a simpler model description for the sensorimotor module, similar to Bogacz et al. (2006), given that only behavioural data are available. However, it should be noted that the biophysical model parameters are constrained by neurobiological properties (e.g. NMDA receptor mediated gating variable dynamics) – hence there were not many free parameters in the model. Further, unlike models as in Bogacz et al. (2006), biologically based model can provide a testbed for future studies using neurophysiological recordings within similar experimental task paradigm. This is important to eventually tease apart the various model types (O’Connell,

Shadlen, Wong-Lin & Kelly, TINS, 2018). Based on this, we have now added a paragraph in Discussion, 5th paragraph:

> *"This model is a variant of the model developed in our previous work (Atiya et al., 2019), which was inspired by neurophysiological recordings from brain regions encoding decision confidence or, reciprocally, decision uncertainty (Kepecs et al., 2008; Murphy et al., 2015). In this work, we have reduced the uncertainty-monitoring module of the original model to lay bare its essential functions. In addition to the similarity of the neural profile of our model's uncertainty-encoding population (Figure 6) to existing recordings (Kepecs et al., 2008; Murphy et al., 2015), this reduced model accounts for some of the main characteristics of decision uncertainty (Kepecs et al., 2008; Kiani et al., 2014; Sanders et al., 2016) (Figure 7). It should be noted that this reduced implementation of decision uncertainty could arguably be less neurobiologically plausible compared to the previous model, as the latter involves a canonical cortical column structure - inhibitory-excitatory pair of neural populations (Wilson & Cowan, 1972; Douglas & Martin, 2004). Importantly, we have not changed how the other (i.e. sensorimotor and motor) modules are described, which can serve as further validation of previous modelling work (Atiya et al., 2019). In particular, the sensorimotor module is based on our previous mean-field model (Wong & Wang, 2006) with biologically derived variables. Therefore, the model can be tested in future studies using neurophysiological recordings within similar experimental task paradigms to allow the distinguishing of different models (O'Connell et al., 2018). It should also be noted that such reductions of biophysical realisations of accumulate-to-bound models (Wang, 2012) can be linked back to simpler models of decision making (Ratcliff, 1978; Usher & McLelland, 2001; Bogacz et al., 2006; Wong & Wang, 2006; Roxin & Ledberg, 2008)."*

Finally, we have also made changes to the table of model parameters. Specifically, we now clarify which model parameters have been modified to account for the current behavioural data (free parameters). Notably, there are only 6 free parameters. The remaining parameters are based directly on Wong and Wang (2006) and Atiya et al. (2019). We have also added a 'Description' column. As such, we have included the updated table below:

Parameter	Description	Value
τ_S	Sensorimotor time constant	100ms
τ_h	Motor time constant	50ms
t_{mc}	Uncertainty population time constant	150 ms
a	Input-output function parameter	270 (V nC)$^{-1}$
b	Input-output function parameter	108 Hz
d	Input-output function parameter	0.154 s
I_0	External tonic input	0.3255 nA
$J_{N,\,ii}$	Self-excitation strength	0.248 nA
$J_{N,\,ij}$	Inhibition strength (sensorimotor)	0.0497 nA
μ_0	Baseline stimulus input	30 Hz
$J_{A,\,ext}$	External input synaptic strength	0.00052 nA Hz^{-1}
J_{mc0}	Uncertainty feedback strength	0.002
J_{VHU}	External input strength (uncertainty)	10 nA
$J_{N,LR}$	Inhibition strength (motor)	1 nA
$J_{N,RL}$	Inhibition strength (motor)	1 nA
S_{th}	Sensorimotor module threshold	42.5 Hz
H_{th}	Hand module threshold	17.4 Hz
G_S	Sensorimotor input-output gain	1.12 Hz

Table 4. Table of model parameter values. Parameters τ_S , a, b, d, I_0, μ_0, $J_{A,\,ext}$ were directly adapted from [30]. Parameters τ_h , t_{mc} , J_{mc0}, $J_{N,\,ij}$, H_{th} were directly adapted from [28], and parameters J_{VHU}, $J_{N,LR}$, $J_{N,RL}$, G_S, S_{th}, $J_{N,\,ii}$ were tuned to account for the behavioural data.

Finally, while the simulations show that the model (more or less) accounts for the phenomena (but I also see some clear deviations in Fig 3), the paper does not provide a transparent explanation why it does so. How does the uncertainty estimation drive (slow-RT) changes of mind? Lets say the subject experiences uncertainty at time t, why/how does this trigger a change of mind? I suppose it has to do with the input from the uncertainty population to the sensorymotor populations, but why should this reverse the earlier choice rather than amplify it?) Also how does the uncertainty relate to the stimulus coherence (is higher moving-dot coherence generate more or less uncertainty, and why? How does the coherence affect the input into the sensorymotor populations). Showing that the model accounts for the data is good, but it would be even better to provide an explanation why it does so. Also I would suggest, that the authors present in the Introduction, the challenge that the present model needs to clear out (the slow COM in the absence of extra-evidence), so that the readers are more focused on this central aim.

We appreciate the reviewer's comment. We have now addressed this by describing the mechanism more clearly in the subsection 'Model's high uncertainty is associated with changes-of-mind'. Specifically, we have included a systematic stability analysis to help us describe the mechanism by which changes-of-mind are associated with decision uncertainty. The new section reads (at the end of the Results section, with Figure 10 in the revised manuscript):

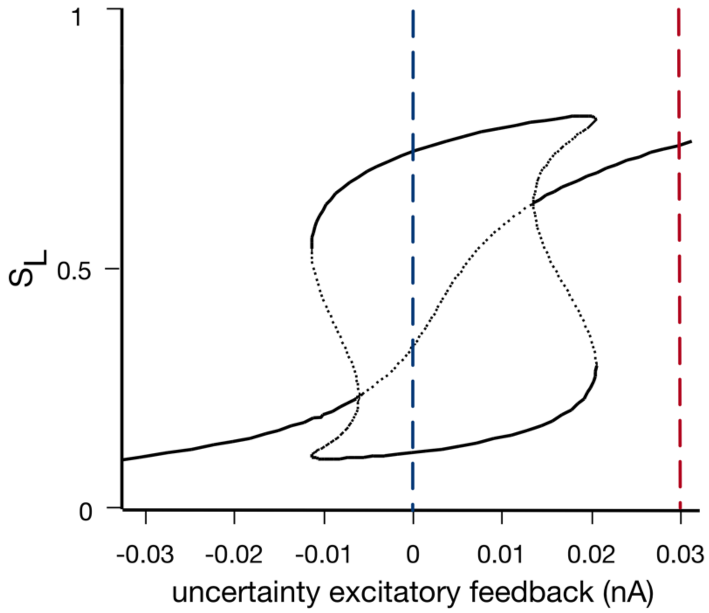

Figure 10. *Bifurcation diagram of the value of the synaptic gating variable S_L (corresponding to the left-selective sensorimotor population) with respect to the magnitude of uncertainty excitatory feedback (at zero coherence level). No or low uncertainty feedback yields two stable steady states (black solid lines) and one unstable steady state (black dotted lines). This forms a "winner-take-all" regime (blue dashed line). In contrast, high uncertainty feedback (around 0.03) yields only one stable steady state (red dashed line).*

"First, we analysed the mean decision uncertainty level (see Methods) as a function of task difficulty (i.e. coherence level) separately for change-of-mind and non-change-of-mind trials (Figure 9a). As previously mentioned, on average, uncertainty levels are higher in the case of change-of-mind trials compared to non-change-of-mind trials (Figure 6, middle panel), regardless of the outcome of the trial (i.e. correct or error). During such trials, we observed longer initial response times (Figure 8d), which allows the uncertainty-monitoring population more time to integrate the summed input from the sensorimotor populations, leading to increased greater neural activity (uncertainty levels), and larger total excitatory feedback to the sensorimotor populations. Interestingly, when sorting the simulated trials based on tertiles of decision uncertainty levels; the probability of a change-of-mind was shown to be highly dependent on the decision uncertainty level (Figure 9b). Hence, our model suggests that changes-of-mind are strongly associated with high decision uncertainty.

To further explain the effect of uncertainty on the dynamics of change-of-mind behaviour in our model, we performed a systematic stability analysis of the value of the population-averaged synaptic gating variable S_L (corresponding to the left-selective sensorimotor population, from Eq. 5) with respect to magnitude of uncertainty feedback (Figure 10) - for simplicity, we set the coherence to be zero for this analysis (see Wong & Wang (2006)).

During the time-course of a change-of-mind trial, the activity of the uncertainty monitoring population gradually increases from zero (Figure 6, red traces). Early in the trial, uncertainty level is close to zero. This leads to low uncertainty excitatory feedback (Figure 10, blue dashed line), and therefore, the network maintains a "winner-take-all" regime with two stable steady states corresponding to two decision states (i.e. Left or Right). As the uncertainty excitatory feedback increases later in the trial, a single

stable steady state appears, which corresponds to indecision (Figure 10, region around the red dashed line). However, since the uncertainty feedback is only transient (Figure 6), the network eventually returns to the initial configuration with two stable steady states (Figure 10, blue dashed line). It should be noted that recurrent network reverberation (Wong & Wang, 2006) allows neural integration even in the absence of a stimulus. Additionally, due to the stochastic nature of the sensory integration, in cases where the choice ends up being different from the initial choice (i.e. an initially "losing" neural population "wins" later in the trial), a change-of-mind occurs (see Atiya et al. (2019) for detailed discussion)."

Specific comments

p. 3. Why is the 1-population implementation less plausible?

We now address this in the Discussion, 5th paragraph:
"It should be noted that this simplified implementation of decision uncertainty could arguably be less neurobiologically plausible compared to the previous model, as the latter involves a canonical cortical column structure - inhibitory-excitatory pair of neural populations (Douglas & Martin, Annu. Rev. Neurosci., 2004; Wilson & Cowan, 1972)."

p. 3 Bottom: eliminate "more" in "more simplified"; change "could still replicate" to "replicates", etc.

Done.

p. 4. Explain in main text how the COM is identified in the data

We appreciate the reviewer's comment. The definition of a change-of-mind is included in the Methods section, Subsection Data Analysis:

> *"A trial was labelled as a change-of-mind trial if a response trajectory was extended for more than 100 pixels (in the x-direction) to the side of the screen with the unchosen option. However, deviations which could have resulted from erratic movements in the early stages of response were ignored: if the threshold of 100 pixels in the horizontal direction was crossed in the bottom 10% of the response area, the trial was not labelled a change-of-mind."*

Fig. 1 (Model) is not very effective. The sensory and hand modelus should be arranged vertically with arrows from one to the other.

We appreciate the reviewer's suggestion. Figure 3a is now redrawn in the revised manuscript. We have also included the new redesigned architecture figure below:

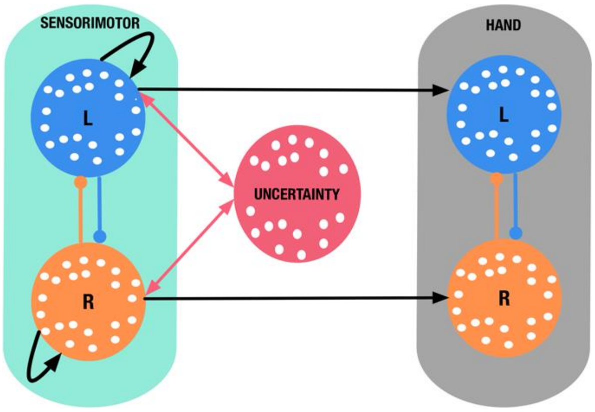

Fig. 5. Is also not very clear. Is this a simulation or an experimental data pattern? If its a simulation one needs more details.

We have now moved this Figure to 6. We now clearly refer to it when mentioning the simulation of the model (Second paragraph, page 7):

> *'We simulated this model (Figure 6) and we observed a phasic neural activity profile for the uncertainty-monitoring population (Figure 6, red activity traces) that is reminiscent of neural recordings from regions or neurons that encode decision uncertainty (Kepecs et al., 2008; Murphy et al., 2015)'.*

References

Bogacz R, Brown E, Moehlis J, Holmes P, Cohen JD. (2006). The physics of optimal decision making: a formal analysis of models of performance in two-alternative forced-choice tasks. Psychol Rev., 113(4):700-65.

Botvinick MM, Braver TS, Barch DM, Carter CS, Cohen JD. (2001). Conflict monitoring and cognitive control. Psychol Rev, 108: 624-52.

Bronfman ZZ, Brezis N, Moran R, Tsetsos K, Donner T, Usher M. (2015). Decisions reduce sensitivity to subsequent information. Proc. R. Soc. B.

Rabbitt, P. (1966). Errors and error correction in choice–response tasks. Journal of Experimental Psychology, 71 , 264–272.

Rabbitt, P. (1967). Time to detect errors as a function of factors affecting choice-response time. Acta psychologica, 27 , 131–142.

Rabbitt, P. (1968). Three kinds of error-signalling responses in a serial choice task. Quarterly Journal of Experimental Psychology, 20 , 179–188.

Rabbitt, P. (1969). Psychological refractory delay and response-stimulus interval duration in serial, choice-response tasks. Acta Psychologica, 30 , 195–219.

Rabbitt, P. (2002). Consciousness is slower than you think. The Quarterly Journal of Experimental Psychology Section A, 55 , 1081–1092.

Rabbitt, P., Cumming, G., & Vyas, S. (1978). Some errors of perceptual analysis in visual search can be detected and corrected. The Quarterly Journal of Experimental Psychology, 30 , 319–332.

Rabbitt, P., & Rodgers, B. (1977). What does a man do after he makes an error? An analysis of response programming. Quarterly Journal of Experimental Psychology, 29 , 727–743.

Rabbitt, P., & Vyas, S. (1981). Processing a display even after you make a response to it. How perceptual errors can be corrected. The Quarterly Journal of Experimental Psychology Section A, 33 , 223–239.

Finally, we thank the reviewer again for bringing these relevant studies to our attention. We highlighted the studies that have been discussed in the revised manuscript in **black bold text.**
